# *18S rRNA* is a reliable normalisation gene for real time PCR based on influenza virus infected cells

**DOI:** 10.1186/1743-422X-9-230

**Published:** 2012-10-08

**Authors:** Suresh V Kuchipudi, Meenu Tellabati, Rahul K Nelli, Gavin A White, Belinda Baquero Perez, Sujith Sebastian, Marek J Slomka, Sharon M Brookes, Ian H Brown, Stephen P Dunham, Kin-Chow Chang

**Affiliations:** 1 School of Veterinary Medicine and Science, University of Nottingham, Sutton Bonington Campus, College Road, Loughborough, Leicestershire LE12 5RD, UK; 2 School of Biosciences, University of Nottingham, Sutton Bonington Campus, College Road,, Loughborough, Leicestershire, LE12 5RD, UK; 3 Virology department, Animal Health and Veterinary Laboratories Agency, Weybridge, Addlestone, UK

**Keywords:** Reference gene, Housekeeping gene, qRT-PCR, Data normalisation, Influenza A viruses, H5N1, H1N1, H2N3

## Abstract

**Background:**

One requisite of quantitative reverse transcription PCR (qRT-PCR) is to normalise the data with an internal reference gene that is invariant regardless of treatment, such as virus infection. Several studies have found variability in the expression of commonly used housekeeping genes, such as beta-actin (*ACTB*) and glyceraldehyde-3-phosphate dehydrogenase (*GAPDH*), under different experimental settings. However, *ACTB* and *GAPDH* remain widely used in the studies of host gene response to virus infections, including influenza viruses. To date no detailed study has been described that compares the suitability of commonly used housekeeping genes in influenza virus infections. The present study evaluated several commonly used housekeeping genes [*ACTB*, *GAPDH*, 18S ribosomal RNA (*18S rRNA*), ATP synthase, H+ transporting, mitochondrial F1 complex, beta polypeptide (*ATP5B*) and ATP synthase, H+ transporting, mitochondrial Fo complex, subunit C1 (subunit 9) (*ATP5G1*)] to identify the most stably expressed gene in human, pig, chicken and duck cells infected with a range of influenza A virus subtypes.

**Results:**

The relative expression stability of commonly used housekeeping genes were determined in primary human bronchial epithelial cells (HBECs), pig tracheal epithelial cells (PTECs), and chicken and duck primary lung-derived cells infected with five influenza A virus subtypes. Analysis of qRT-PCR data from virus and mock infected cells using *NormFinder* and *BestKeeper* software programmes found that *18S rRNA* was the most stable gene in HBECs, PTECs and avian lung cells.

**Conclusions:**

Based on the presented data from cell culture models (HBECs, PTECs, chicken and duck lung cells) infected with a range of influenza viruses, we found that *18S rRNA* is the most stable reference gene for normalising qRT-PCR data. Expression levels of the other housekeeping genes evaluated in this study (including *ACTB* and GPADH) were highly affected by influenza virus infection and hence are not reliable as reference genes for RNA normalisation.

## Background

Real-time PCR or quantitative reverse transcription PCR (qRT-PCR) is widely used to quantify changes in messenger RNA (mRNA) levels. In many cases, it is the only reliable and sensitive method of quantification of mRNA levels of low copy number targets [1]. There are several advantages of qRT-PCR over other conventional methods for measuring mRNA levels such as accurate quantification, high sensitivity, large dynamic range, and potential for high throughput analysis [1]. However, the following factors need to be properly addressed to avoid erroneous qRT-PCR results: variability of RNA quantification and dispensing, and different efficiencies of reverse transcription (RT) and PCR [2]. Consequently, it is important to choose a reliable normalisation method to take into account errors introduced by these factors.

A widely used method for normalisation involves measuring the expression of an internal reference or "housekeeping" gene, which takes into account the potential error of RNA/cDNA loading, and variation of reverse transcription efficiency [3]. An ideal reference/ housekeeping gene should be stably expressed across samples from different tissues, developmental stages, and experimental conditions. However, there is no single gene that can satisfy all these criteria. Nevertheless, it is important to ensure that the expression of a reference gene used in a particular experiment is not adversely affected by the treatment. Normalisation of gene expression based on a varying reference gene is likely to produce misleading results [4]. Three commonly used reference genes for normalising qRT-PCR data are *ACTB*, *GAPDH*, and *18S rRNA*[5]. However, several studies have reported that levels of *ACTB* and *GAPDH* are highly variable among cell types, during cell differentiation and in cancers [6-10].

Virus infection of cells leads to a general inhibition of cellular macromolecular synthesis that is referred to as shut-off [11] and causes changes in global gene expression. Therefore, it is essential to validate reference genes to ensure their suitability for a specific experiment involving a particular virus and cell type [12]. Expression of many genes including *ACTB* are significantly altered in human cell lines following infection with cytomegalovirus, human herpes virus-6, camelpox virus, severe acute respiratory syndrome (SARS) coronavirus and yellow fever virus [13]. Many studies determined the reliability of housekeeping genes in different cells infected with a range of different viruses [13-16] , however to date no detailed study has been carried out to demonstrate suitability of reference genes that could be used in influenza A virus infected avian and mammalian cells.

Despite their reported instability and unsuitability as reference genes, *ACTB*[17-19] and *GAPDH*[20,21] remain widely used for normalising qRT-PCR data in influenza infection studies. We examined the stability of *ACTB*, *GAPDH*, *18S rRNA*, *ATP5B* and *ATP5G1* to identify a suitable housekeeping gene for qRT-PCR normalisation of data from primary human bronchial epithelial cells, pig tracheal epithelial cells, chicken and duck lung cells infected with a range of low and high pathogenicity influenza A viruses.

## Results and discussion

RNA expression stability of commonly used reference genes was studied in primary cells from human, pig, chicken and duck at 24h following infection with five influenza A virus subtypes. Expression of *18S rRNA*, *ACTB*, *GAPDH*, *ATP5B*, *ATP5G1* were compared using *BestKeeper* and *NormFinder* software programmes in virus and mock infected samples.

Raw crossing point (Cp) values from each of the virus and mock infected samples (n=6) were used to calculate standard deviation [SD (± Cp)] for all the reference genes using *BestKeeper* software (Table 1). Separate analyses were carried out for each cell type. Based on the variation (SD) in expression, *18S rRNA* was the most stable among all the genes tested in HBECs, PTECs, and chicken and duck lung cells 24h following infection with various influenza virus subtypes (Table 1). Expression stability of remaining reference genes varied between virus treatments and species. Further pair-wise correlation and regression analysis was carried out using *BestKeeper* software to calculate the correlation between the expression of each of the candidate reference genes and the *BestKeeper* index. Cp values for all three viruses and mock infected samples (n=12) were used for this analysis and separate analyses were carried out for each cell type. In all four cell types, a strong significant correlation (0.843< r > 0.962) was detected between *18S rRNA* gene expression and the *BestKeeper* index (p<0.01) (Table 2) compared with the other genes. *GAPDH* was the next best gene based on the correlation coefficient values in HBECs, PTECs and duck lung cells (0.792< r > 0.871). For chicken lung cells *ACTB* was the second best reference gene (r = 0.845).

**Table 1:** ***Bestkeeper *analysis of housekeeping genes showing variation in gene expression**

	**Standard deviation [± Cp]**			
**HBECs**	***18S rRNA***	***ACTB***	***GAPDH***	*ATP5B*/ ATP5G1*
H5N1 tyTR05	0.474	2.484	1.196	1.620
Swine H1N1	0.375	0.484	0.465	0.413
USSR huH1N1	0.462	1.046	0.740	0.662
Average	0.437	1.338	0.800	0.899
**PTECs**				
Swine H1N1	0.265	0.354	0.344	0.413
H5N1 tyEng91	0.090	0.330	0.310	0.140
H5N1 tyTR05	0.100	0.420	0.240	0.450
Average	0.152	0.368	0.298	0.334
**Chicken lung cells**				
H5N1 tyEng91	0.160	0.510	0.480	-
H5N1 tyTR05	0.140	0.230	0.260	-
Avian H2N3	0.200	0.230	0.200	-
Average	0.167	0.323	0.313	-
**Duck lung cells**				
H5N1 tyEng91	0.380	0.480	0.690	-
H5N1 tyTR05	0.160	0.660	1.290	-
Avian H2N3	0.110	0.190	0.430	-
Average	0.217	0.443	0.803	-

Bestkeeper analysis (n=6) of standard deviation (± Cp) showed *18S rRNA* is the most stable gene in all the four cell types at 24h post-infection with the influenza virus subtypes used in this study. *ATP5B and *ATP5G1* were included in HBECs and PTECs analysis respectively.

**Table 2:** ***Bestkeeper *correlation and regression analysis of housekeeping genes**

	***18S rRNA***	***GAPDH***	***ACTB***	***ATP5B*/ *ATP5G1*cp**
	**vs.**	**vs.**	**vs.**	**vs.**
	***BestKeeper***	***BestKeeper***	***BestKeeper***	***BestKeeper***
	**Coefficient of correlation [r]**			
**HBECs**	0.962	0.871	0.709	0.543
**PTECs**	0.843	0.743	0.741	0.73
**Chicken lung cells**	0.944	0.832	0.845	-
**Duck lung cells**	0.836	0.792	0.542	-

Pairwise correlation and regression analysis using *Bestkeeper* correlation analysis (n=12) found *18S rRNA* as the most suitable housekeeping gene. Note that highest correlation between *18S rRNA* and the *BestKeeper* index (p<0.01) was found in all the four cell types used. *ATP5B and *ATP5G1*were included in HBECs and PTECs analysis respectively.

**Figure 1:**
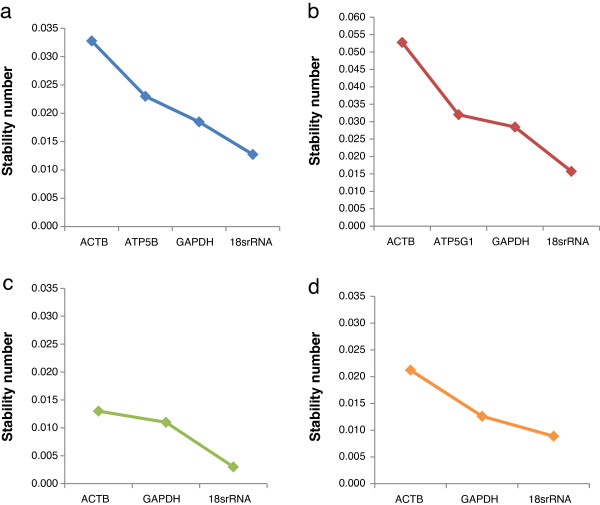
***NormFinder *analysis of housekeeping genes in human (a), pig (b), chicken (c) and duck (d) cells showing stability numbers, which are a measure of the systematic error introduced by each of the reference genes when used to normalise the qRT-PCR data.** In all of the four cell types, *18S rRNA* was the best reference gene as evidenced by the lowest stability numbers (0.003 to 0.016) among all of the genes compared. *GAPDH* was the second gene of choice for all the four host species. *ACTB* was the most unstable gene in all four host cell types. Data represent stability numbers which were calculated by combining intra- and inter- group variations (n=12).

*NormFinder* software calculates a stability number from the intra- and inter-group variations which represent a measure of the systematic error introduced by each of the reference genes when used to normalise the data. *NormFinder* analysis of reference genes in HBECs (Figure 1a), PTECs (Figure 1b), chicken (Figure 1c) and duck (Figure 1d) lung cells also indicated that *18S rRNA* was the best among the housekeeping genes comparison, with lowest stability numbers ranging from 0.003 to 0.016. Based on the *NormFinder* stability numbers, *GAPDH* was the second best gene, while *ACTB* was the most unstable gene in all the four cell types.

BestKeeper software is robust against sampling errors but it requires that none of the genes analyzed are co-regulated. In contrast NormFinder is less affected by co-regulation of genes but could be sensitive to sampling errors [22]. Hence, the use of both software programmes in the present study provided a robust and accurate analysis of the expression stability of the candidate housekeeping genes.

In summary we concluded that*18S rRNA* is a suitable housekeeping gene, while *ACTB* and *GAPDH* are not as reliable for normalising qRT-PCR data from influenza virus infected HBECs, PTECs, chicken and duck cells.

Ribosomal RNA, the central component of the ribosome is an abundant and one of the most conserved genes in all cells [23]. We found that expression of *18S rRNA* was least affected by the infection of influenza A virus subtypes in all the cell types used in this study. Several studies have also recommended ribosomal RNA as the best choice as a reference gene compared with other genes in a variety of cell culture systems [24-27].

A technical limitation of using *18S rRNA* as a normaliser is that random primers must be used for cDNA synthesis rather than oligo-(dT) since rRNA does not contain a poly-A tail. Use of oligo-(dT) has been suggested as being preferable over random oligomers for cDNA synthesis in order to avoid multiple initiations and to obtain a single initiation event per individual mRNA [28]. To overcome this problem a co-application reverse transcription (Co–RT) with *18S rRNA* sequence-specific primer combined with oligo-(dT) reaction could be used to increase the sensitivity and improve accuracy of reverse transcription [29]. Another criticism of using *18S rRNA* for normalising mRNA expression is that the ribosomal fraction may not truly represent the overall cellular mRNA population [29]. However, influenza virus infection inhibits cellular macromolecular synthesis (shut-off) and causes global gene expression changes [30-35] affecting mRNA levels of many genes. In our laboratory, we performed transcriptome analyses of influenza virus infected human, pig, chicken and duck cells using DNA-microarrays, and found that many genes including *ACTB* ,*GAPDH,* SDH1, *EEF1G, PPI, TBP* and *ATP5B* to be differentially regulated (data not shown). Despite the perceived disadvantages, owing to its consistency and stability of expression in influenza virus infected cells, *18S rRNA* is the most appropriate gene to be used as a reference gene.

Tracheo-bronchial epithelial cells from human, pig and lung fibroblasts from chicken and ducks were used in the present study. It is likely that the expression stability of *18S rRNA* could be different among other cells and host species. However, previous studies found that the*18S rRNA* gene was the most stable gene in resting and polyclonal T cell activated human peripheral blood mononuclear cells (PBMCs), [10], and hepatic cells in chicken [36,37] and geese [38].

A recent study comparing the expression of 11 housekeeping genes including *ACTB* and *GAPDH* found that *ACTB* and ribosomal protein L4 (RPL4) were the most stable in H5N1virus infected chicken embryo fibroblasts (CEF) cells and *GAPDH* as the most stable gene in normal CEF cells [39]. However, this study did not include *18S rRNA* for comparison, it used only one H5N1 virus isolate and the data were analysed using the GeNorm software programme [39]. In the present study the data were subjected to robust analyses with two different software programmes that are based on different algorithms. Furthermore, *NormFinder* software used in this study provides results consistent with *GeNorm* using higher sample sizes [40].

Other strategies such as normalising against total RNA [1] [for example ribogreen (molecular probes), LabChip (Agilent)] and genomic DNA [41] have also been used. A major drawback of normalising against total RNA is that it doesn’t account for the inherent variation in the reverse transcription or PCR reactions [42]. There is also a major problem for normalising against genomic DNA, because the extraction rates of RNA and DNA may vary between different samples, with yields of DNA often being low. The presence of variable haplotypes in certain tumour cells [1] and multiple copies of particular loci in replicating bacteria compared to non replicating bacteria [43] are some of the additional problems of normalising against genomic DNA. Use of multiple reference genes rather than one has also been suggested as a robust method for providing accurate normalisation [44]. However, it may not always be feasible to use multiple reference genes due to limitations of the sample availability, cost and even when using multiple reference genes, accuracy remains dependent on the variability of the chosen reference genes [1]. Although these methods are not mutually exclusive, normalising qRT-PCR data using a single internal reference gene continues to be the most widely used method .To conclude, in this study for influenza A infected host cells the most suitable housekeeping gene appears to be *18S rRNA*.

## Methods

### Viruses and cell cultures

A H2N3 low pathogenicity avian influenza virus (LPAIV) (A/mallard duck/England/7277/06), a classical swine H1N1 (A/sw/Iowa/15/30), a human H1N1 (A/USSR/77), a classical H5N1 highly pathogenic avian influenza virus (HPAIV) (A/turkey/England/50-92/91, hereafter referred to as H5N1 tyEng91) and a contemporary H5N1 Eurasian lineage (clade 2.2.1) HPAIV (A/turkey/Turkey/1/05, hereafter referred to as H5N1 tyTR05) were used in this study. All the viruses were grown in 10-day-old embryonated chicken eggs by allantoic inoculation. Viruses were titrated using Madin-Darby canine kidney (MDCK) cells by a previously described immuno-cytochemical focus assay [45].

Primary human bronchial epithelial cells (HBECs) (CC-2540) from Lonza UK were used. Pig tracheal epithelial cells (PTECs) were isolated from stripped tracheo-bronchial mucosae from 3- to 4-month-old euthanized pigs as previously described [46]. Briefly, washed minced mucosae were incubated at 4°C overnight with 0.06U/ml pronase (Sigma) in 1:1 DMEM:F-12 medium. Supernatant containing cells were centrifuged and washed in DMEM-Glutamax. Both HBEC and PTECs were cultured in bronchial epithelial growth medium (BEGM, CC-3170, Lonza UK). Primary lung cell cultures were extracted from lungs of euthanized 4-week-old broiler chickens and 6-week-old Pekin ducks and were grown in Dulbecco's modified Eagle's medium (DMEM, Invitrogen Ltd., Paisley, UK) with antibiotics using a method that we previously described [45].

### Virus infection of cells

All cells were grown in 6 well cell culture plates (Corning) and three wells were used for each cell and virus type. HBECs and PTECs were cultured in serum-free BEGM, chicken and duck lung cells in DMEM : Ham’s F12 (1:1), containing 2% Ultroser G (Pall Biosepra), 50ng/ml TPCK trypsin (Sigma) and 100 U/ml penicillin-100μg/ml streptomycin. All cells were pre-incubated with the virus for 2h to achieve a multiplicity of infection (MOI) of 1.0. Mock infected controls in three wells were performed by adding equivalent amount of PBS instead of virus in medium. The following virus-cell combinations were used for the infection study.

### HBECs

H5N1 tyTR05, USSR huH1N1 and swine H1N1.

### PTECs

H5N1tyEng91, H5N1 tyTR05 and swine H1N1.

### Chicken and duck cells

H5N1 tyEng91, H5N1 tyTR05 and avian H2N3.

After 2h, cells were rinsed three times with PBS and incubated in fresh medium until harvest at 24h post-infection (PI). Virus infection of cells was confirmed by immuno-chemical staining using a murine monoclonal antibody to influenza nucleoprotein (Abcam) with a DAKO Envision system as previously described [45] (Data not shown).

### Extraction of total RNA

Total RNA from cells was extracted using RNeasy plus - QIAshredder Kit (Qiagen) following the manufacturer’s instructions. The concentration of extracted RNA in samples was determined using UV absorption with a NanoDrop1000 spectrophotometer (Thermo Scientific) and the quality of RNA was assessed using Agilent RNA 6000 nano kit (Agilent) following the manufacturer’s instructions.

### cDNA synthesis

A two step qRT-PCR assay was used in which a first strand cDNA was synthesized with10μg of the total RNA sample using random primers and Superscript III First-strand synthesis system (Invitrogen) following the manufacturer’s instructions.

### Primers

Expression of *ACTB*, *GAPDH* and *18S rRNA* were analyzed in virus and mock infected cells from all of the four species. In addition *ATP5B* and *ATP5G1* were also included for human and pig analysis. Commercially available primers obtained from Primer Design Ltd (UK) were used for human and pig genes and hence the sequences are not available. Primers for chicken genes were designed using Primer express software version 2.0 (Applied Biosystems) and the same primers were used for both chicken and duck samples. Sequence details for the chicken primers: *ACTB-* TGCTGCGCTCGTTGTTGA (Fwd), TCGTCCCCGGCGAAA (Rev), *GAPDH* -GAAGCTTACTGGAATGGCTTTCC (Fwd), CGGCAGGTCAGGTCAACAA (Rev), and *18S rRNA-* TGTGCCGCTAGAGGTGAAATT (Fwd), TGGCAAATGCTTTCGCTTT (Rev). Chicken primers were provided by Sigma (UK).

### Quantitative PCR

Quantitative RT-PCR for the relative expression analysis of selected genes was carried out using the *LightCycler* ® 480 SYBR green master mix (Roche) and all the reactions were carried out using the *LightCycler*® 480 (Roche). A master mix was prepared for each target gene comprising 10μl of SYBR Green master mix, 0.8μl each of forward and reverse primers (900nM) and 3.6 μl of nuclease free water. Five micro litres of cDNA diluted at 1:25 was used per reaction in a total reaction volume of 20μl. PCR cycling parameters were as follows; denaturation at 95°C for 10min followed by 45 cycles of 95°C for 15s, 60°C for 30s, 72°C for 1s followed by cooling at 40°C for 10s. Product specificity was evaluated by melting curves. Each total RNA sample was amplified in triplicate and the mean values were used for further analysis. Crossing point (Cp) values were calculated in the absolute quantification mode using the second derivative method. Serial dilutions of a pooled cDNA sample for each cell type (combination of virus and mock infected samples) were used to plot a standard curve. Using the slope of the standard curve, PCR efficiencies were calculated using the formula, Efficiency = −1+10^(−1/slope)^. Raw Cp values were used for *BestKeeper* analysis while expression values generated by the relative standard curve method were used for *NormFinder* analysis. PCR efficiencies of all the genes tested ranged between 90-100%.

### Determination of expression stability of reference genes

*BestKeeper* and *NormFinder* software programmes which utilise pair-wise comparison and model based approaches respectively were used to determine the stability of each of the housekeeping genes in the various virus and mock infected groups following the developer’s instructions.

*BestKeeper* software [47] was used initially to calculate standard deviation (SD) (± Cp) based on the raw Cp values from each of the virus infected and control samples (n=6) for all of the reference genes. SD values provide a measure of the variation and are used to determine the expression stability of candidate reference genes. Further pair-wise correlation- regression analysis was also carried out with all the virus and mock infected Cp values for each cell type (n=12). *BestKeeper* combines all the reference genes for a sample into *BestKeeper* index using the geometric mean of Cp values of each of the candidate gene. The software then undertakes pair-wise correlation analyses, to determine the relationship between each gene by assigning each gene combination a Pearson correlation coefficient (r) and a probability (p) value. The software combines highly correlated genes into an index and the software then compares the correlation between each gene pair and the *BestKeeper* index to calculate the correlation coefficient (r) value. In a panel of housekeeping genes, the ideal housekeeping gene is the one with the highest correlation coefficient (r).

*NormFinder* software [22] uses a model based evaluation strategy where the software first calculates intra- and inter-group variation and combines the two into a stability value. The stability value is a combination of the two sources of variation and hence is a practical measure of the systematic error introduced by a gene when used as a housekeeping gene for data normalization. A gene with a low stability number is less likely to introduce systematic error that a gene with a high stability number. Raw non-normalized expression values generated by the standard curve method were used for *NormFinder* analysis (n=12).

## Competing interests

The authors declare no competing interests.

## Authors’ contributions

SVK carried out the statistical analysis of the data, designing of the primers and drafted the manuscript. SVK, MT, GAW and RKN carried out the qRT-PCR assays. GAW, RKN performed human and pig cell infections, RNA extractions and cDNA synthesis. BB and SS performed chicken and duck primary cell cultures, virus infections, RNA extractions and cDNA synthesis. SPD, MJS and SMB conducted the H5N1 infection studies in high containment facilities (ACDP3/SAPO4). SPD, IHB participated in the study design and coordination. SVK and KCC conceived the study, participated in the design and helped to draft the manuscript. All authors read, provided input and approved the final manuscript.
